# Supplementary material for: Iodine staining as a useful probe for distinguishing insulin amyloid polymorphs
Source: Sci Rep. 2020 Oct 7;10:16741. doi: 10.1038/s41598-020-73460-y (PMC7542459; doi:10.1038/s41598-020-73460-y)
Supplement: Supplementary file 1 — Supplementary information. [file 41598_2020_73460_MOESM1_ESM.pdf]

Supplementary Information

Iodine staining as a useful probe for distinguishing insulin amyloid polymorphs

Takato Hiramatsu, Naoki Yamamoto, Seongmin Ha, Yuki Masuda, Mitsuru Yasuda, Mika Ishigaki, Keisuke Yuzu, Yukihiro Ozaki & Eri Chatani

Figure S1. Far-UV CD spectra of insulin molecules in a solution state before the fibrillation in the absence or presence of NaCl or SDS.

Figure S2. UV-Vis spectra showing negligible interaction between iodine molecules and native insulin.

Figure S3. UV-Vis spectra their second derivatives of iodine-stained amyloid fibrils showing negligible effects of the presence of salts.

Figure S4. Representative results of curve fitting of the UV-Vis absorption spectra in the seeding reaction.

Figure S5. Representative results of curve fitting of the UV-Vis absorption spectra in the iodine titration experiment.

Figure S6. Images of an unstained spherulite observed by polarization microscopy.

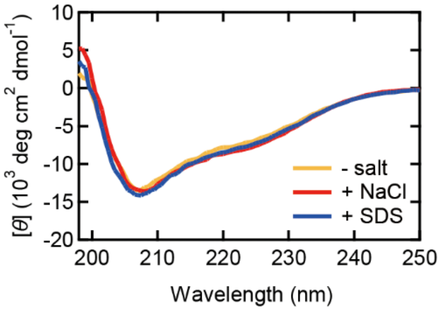

Figure S1. Far-UV CD spectra of insulin molecules in a solution state before the fibrillation in the absence or presence of NaCl or SDS. Insulin solutions with the same compositions as used in the formation of three polymorphs, i.e., 2.0 mg/ml insulin dissolved in 25 mM HCl in the case without any additives (-salt) and with 100 mM NaCl (+NaCl) or 100 μM SDS (+SDS) were subjected to the measurements. Far-UV CD spectral measurements were performed using a CD spectrometer J-1100 (JASCO, Tokyo, Japan). Each sample solution was sealed in a quartz cell with a path length of 0.2 mm and incubated at 65 °C for 5 minutes before the measurement. The measurement was performed at 20 nm/min, and four individual scans were integrated and averaged to obtain a final spectrum. The total time of the measurement was approximately 15 min, within which the shape of spectrum was unchanged.

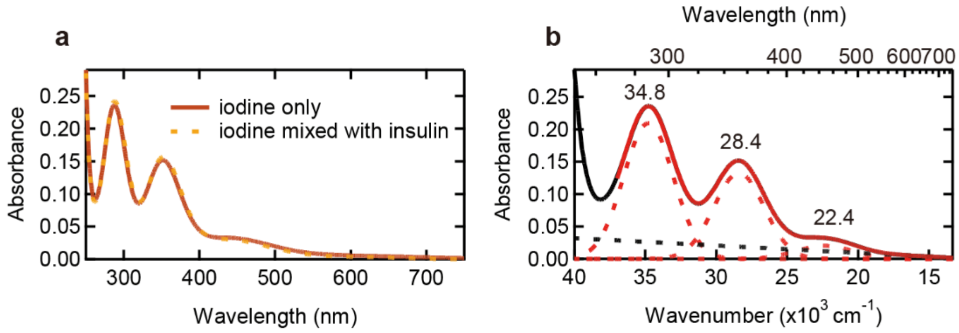

Figure S2. UV-Vis spectra showing negligible interaction between iodine and native insulin. (**a**) UV-Vis spectra of an iodine solution (red line) and that mixed with native insulin (dashed orange line). With regard to the latter spectrum, the spectrum of native insulin was subtracted to show a net absorption spectrum derived from iodine species. The iodine staining was performed under the conditions of 0.3 mM KI and 0.04 mM I_2 in 25 mM HCl, and the concentration of native insulin was 0.25 mg/ml. The dashed orange line was overlapped with the red line, suggesting that there was no significant interaction between iodine and native insulin. (**b**) Result of curve fitting of the spectrum of iodine solution. A black line represents the experimental spectrum, and a red line represents the fitted spectrum using eq. 1 assuming three Gaussian bands (i.e., n = 3). The Gaussian bands and the baselines are represented with dashed red lines and a dashed black line, respectively.

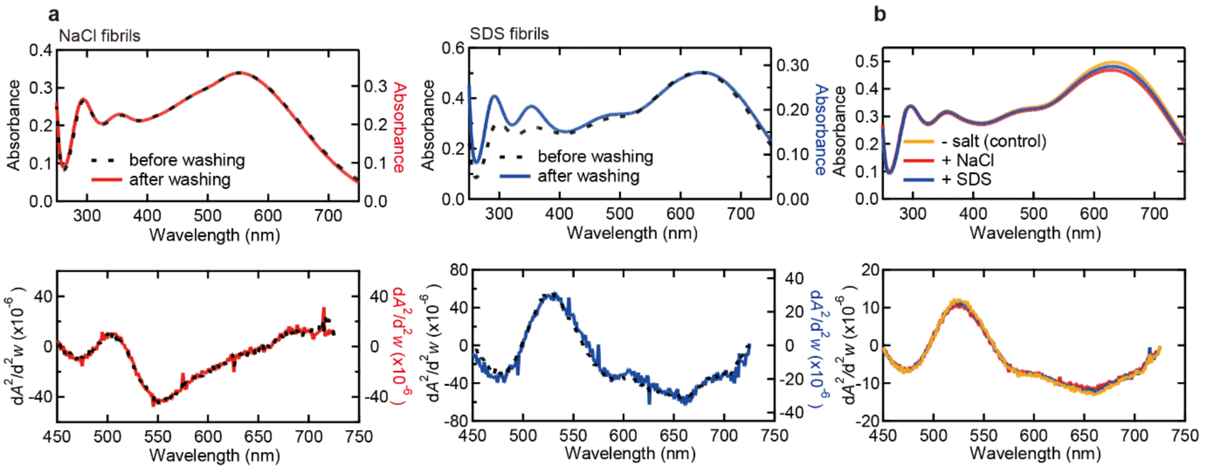

Figure S3. UV-Vis spectra their second derivatives of iodine-stained amyloid fibrils showing negligible effects of the presence of salts. To confirm that the contamination of salt from the parent fibrils does not influence iodine staining, two kinds of tests, i.e., iodine staining of NaCl and SDS fibrils after washing (**a**) and that of no-salt fibrils in the presence of NaCl or SDS (**b**), were carried out. In the former experiment, NaCl and SDS daughter fibrils were centrifuged at 21500 ×g for one hour and the supernatant was replaced with 25 mM HCl. After repeating this washing procedure twice, the samples were stained with iodine. Iodine staining was carried out with the same protocol as that used in Figure 2, assuming that the fibril concentration of the samples did not change before and after washing. However, SDS fibrils hardly precipitated by centrifugation and the yield after washing was low. To compensate for the fibril loss, the spectrum after washing were rescaled by peak intensity when overlaid with that before washing. In the latter experiment, no-salt fibrils were initially prepared, and after the treatment with ultrasonic pulses, NaCl or SDS was added to the fibrils at a final concentration of 5 mM or 5 μM, respectively; these concentrations correspond to those of NaCl or SDS contained in the NaCl/SDS daughter fibrils coming from the parent seed fibrils. After the incubation of the amyloid fibrils at 37 °C for 24 hours, the samples were iodine-stained with the same protocol as that used in Figure 2. As a result, the shapes of the absorption spectra and their second derivatives were unchanged even after the fibril washing or the addition of NaCl or SDS, eliminating the effect of the residual salts on the color formation by iodine staining.

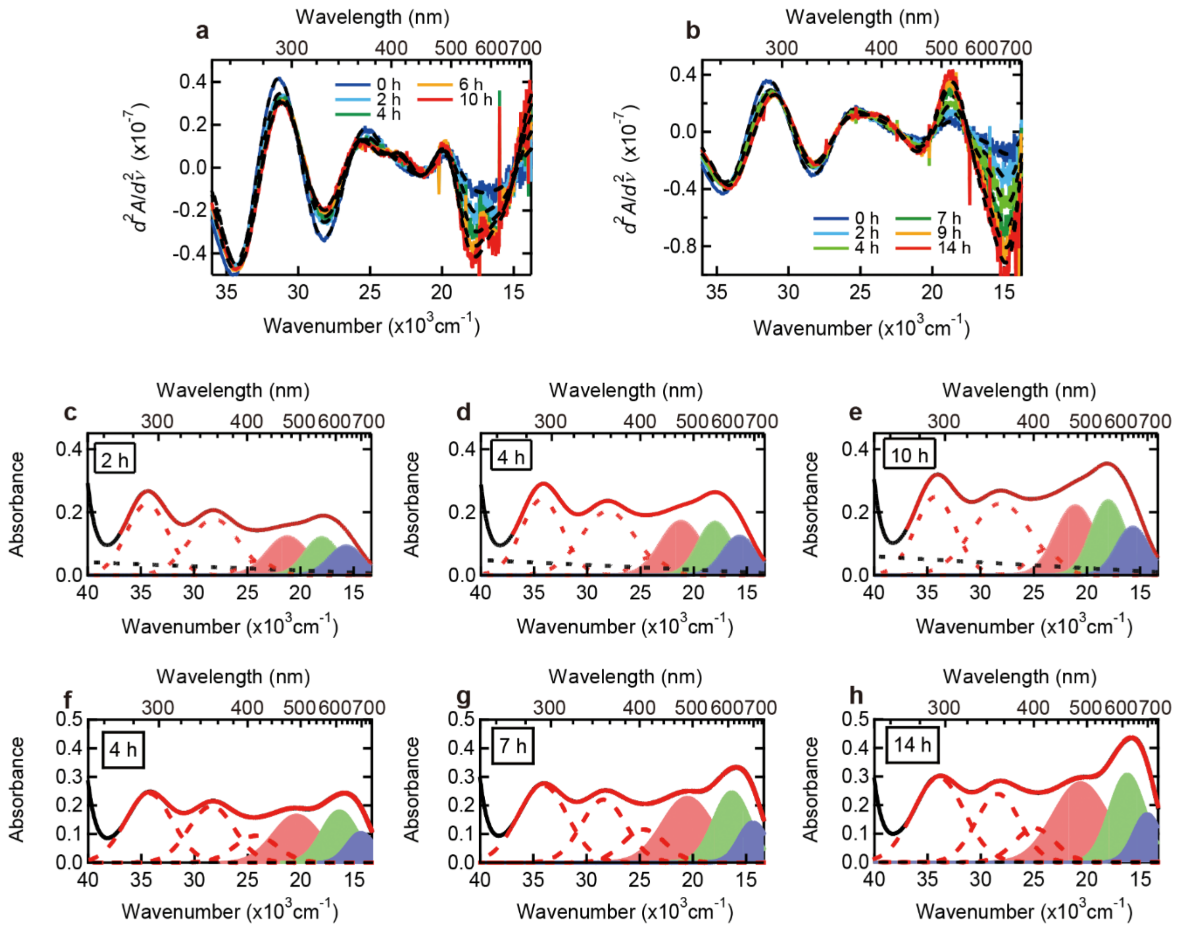

Figure S4. Representative results of curve fitting of the UV-Vis absorption spectra in the seeding reaction. (**a** and **b**) Second derivatives of the absorption spectra of iodine-stained NaCl (**a**) and SDS fibrils (**b**) shown in Fig. 4a and c. Dashed lines are the second derivatives of the fitted spectra obtained by the curve fitting with six Gaussian peaks, which was constructed to assess the validity of the curve fitting. (**c**-**h**) Results of curve fitting of NaCl (**c**-**e**) and SDS fibrils (**f**-**h**) at three representative time points. In each panel, black and red solid lines represent the experimental and the fitted spectra, respectively. Six Gaussian peaks are shown with red dashed lines or filled areas and a baseline is shown with a black dashed line. In performing curve fitting, the position and FWHM of the band II (green filled area) for NaCl fibrils or the band III (blue filled area) for SDS fibrils, the most prominent band among the three bands with the largest minimum in the second derivatives, were fixed to values read from the results of second derivatives.

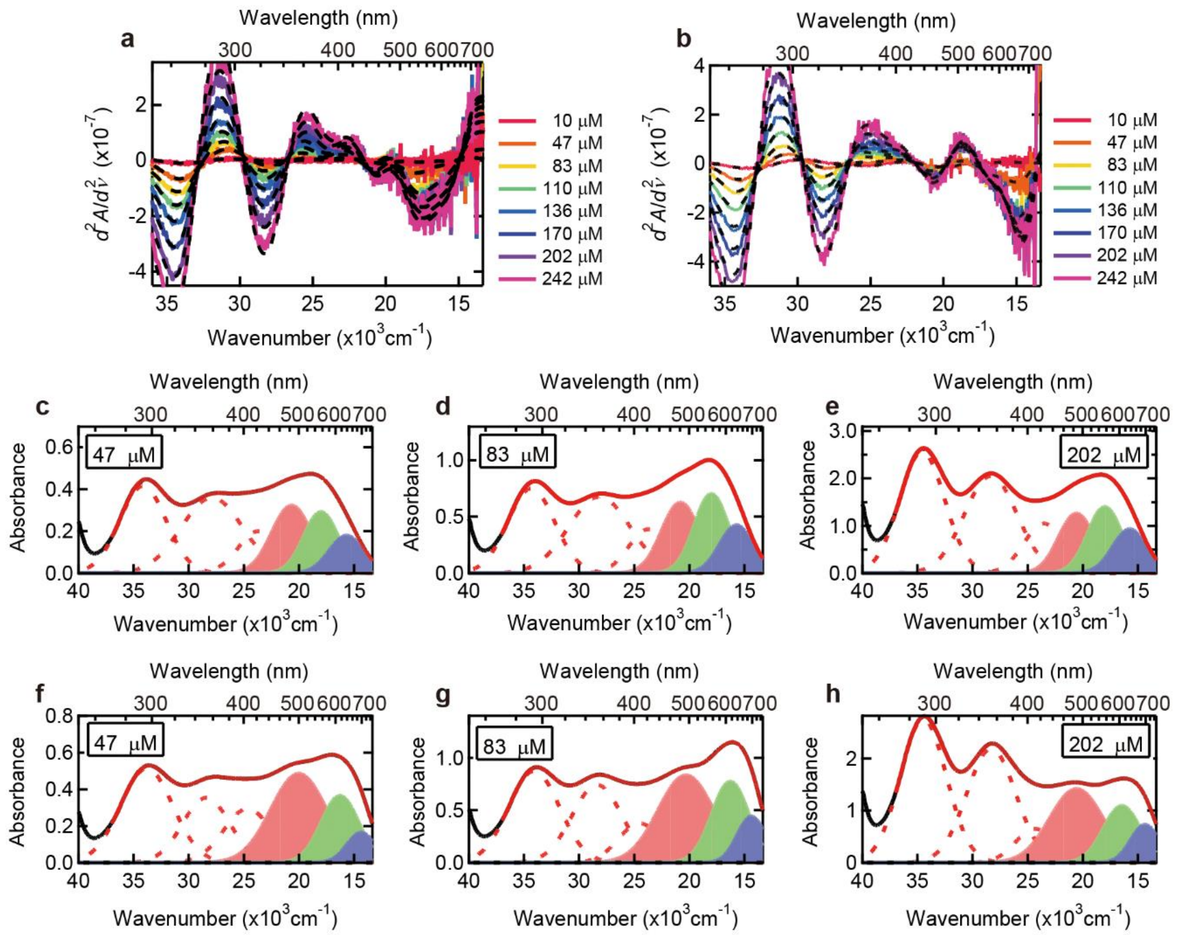

Figure S5. Representative results of curve fitting of the UV-Vis absorption spectra in the iodine titration experiment. (**a** and **b**) Second derivatives of the absorption spectra of iodine-stained NaCl (**a**) and SDS fibrils (**b**) shown in Fig. 6a and b. Dashed lines are the second derivatives of the fitted spectra obtained by the curve fitting with six Gaussian peaks, confirming the consistency of the fitting results. (**c**-**h**) Results of curve fitting of NaCl (**c**-**e**) and SDS fibrils (**f**-**h**) at three representative iodine concentrations. In each panel, black and red solid lines represent the experimental and the fitted spectra, respectively. Six Gaussian peaks are shown with red dashed lines or filled areas and a baseline is shown with a black dashed line. In performing curve fitting, the position of the band II (green filled area) for NaCl-fibrils or the band III (blue filled area) for SDS-fibrils and its FWHM were fixed based on the results of second derivatives.

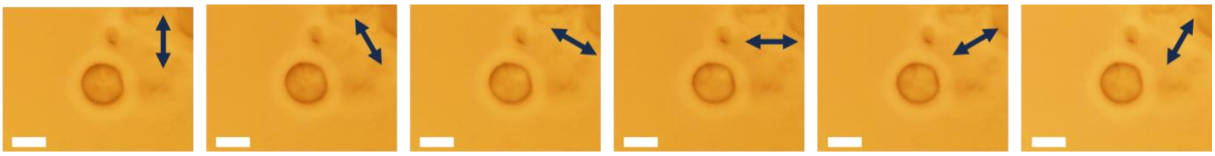

Figure S6. Images of an unstained spherulite observed by polarization microscopy. The direction of the analyzer is represented with a double-headed arrow in the upper right of each image. Scale bars represent 10 μm.
